# Effect of therapeutic exercise on peak oxygen consumption in oncological population: a systematic review with meta-analysis

**DOI:** 10.1007/s00520-024-09004-1

**Published:** 2024-11-13

**Authors:** Alicia del-Rosal-Jurado, Manuel González-Sánchez, Antonio Ignacio Cuesta-Vargas

**Affiliations:** 1 Department of Physiotherapy, Institute of Biomedicine of Málaga (IBIMA), Faculty of Health Sciences, University of Málaga, 29071 Málaga, Spain; 2 School of Clinical Sciences of the Faculty of Health, Queensland University of Technology, Brisbane, QLD 4059 Australia

**Keywords:** Cancer, Neoplasms, Exercise, Respiratory function tests, Cardiorespiratory fitness

## Abstract

**Introduction:**

Cancer is amongst the leading causes of death worldwide. A total of 19.3 million new cases were diagnosed in 2020. Cardiovascular impairment is the second leading cause of death in patients undergoing cancer treatment. By measuring the maximum rate of oxygen consumption (VO_2_max) in cancer patients, it is possible to estimate their cardiorespiratory health. This study aims to show how participants’ VO_2_max increases with a therapeutic physical exercise programme.

**Methods:**

Four databases were used for the literature search. Twenty-eight studies were analysed using the inclusion and exclusion criteria. The quality of these studies was analysed with the PEDRO scale. The structural characteristics of the articles and the study variable (VO_2_max) were studied.

**Results:**

The VO_2_max of the 2558 patients studied presents some variations by gender, intervention and moment of measurement. For male-associated cancer, the increased VO_2_max ranged between 1.5 and 4.2% after the intervention. The increase in the maximum VO_2_max in female-associated cancer was between 0.3 and 53%. Different types of cancer for both genders presented an oscillation between 0.5 and 3.47%.

**Conclusions:**

Therapeutic physical exercise is an efficient intervention to improve the VO_2_max in oncology patients. The results show that a therapeutic physical exercise intervention lasting 12 weeks, 3 days per week with a moderate-vigorous intensity, increases the VO_2_max in oncology patients.

## Introduction

Cancer is amongst the leading causes of death worldwide [1]. A total of 19.3 million new cases were diagnosed in 2020 [1]. Estimates show there may be 30.2 million new cases of cancer by 2040 [1]. Advances in diagnostics and treatment techniques are increasing the number of long-term cancer survivors [2]. Differentiation by sex has caused the level of specificity in the diagnosis and treatment of cancer to increase in recent years. In this sense, there are cancers that, due to the specific anatomy of women, are identified as “female cancers” [3] and, similarly, due to the specific anatomy of men, “male cancers” [4] are also identified. Cancer-related effects can be diminished by therapeutic physical exercise (TPE). High-level physical activity decreases the likelihood of cancer by 27% [5]. Regular physical activity reduces the risk of developing colon cancer, breast cancer and endometrial cancer [6]. TPE is most effective when supervised, in person or online, by a health professional [7]. Cardiorespiratory function is a declining adverse effect of cancer [8]. Cardiovascular impairment is the second leading cause of death in patients undergoing cancer treatment [9]. Cardiorespiratory function is an important predictor factor of health and mortality [10]. By measuring maximum rate of oxygen consumption (VO_2_max) in cancer patients, it is possible to estimate their cardiorespiratory health [2]. Performance of TPE by oncology patients results in changes in vagal tone, leading to an increase in cardiorespiratory fitness and VO2max [9].

As indicated above, there are studies demonstrating the benefits of TPE on cardiorespiratory fitness. There are currently no systematic reviews on the most common tumours and types of training used to increase the VO2max of participants.

This study aims to show how participants’ VO2max increases with a TPE programme.

## Methods

This systematic review was carried out in agreement with the Preferred Reporting Items for Systematic Review and Meta-Analyses (PRISMA) statement and was registered in PROSPERO with reference number CRD42018103258.

### Search strategy

The literature research was performed through the PubMed, Web of Science, PEDro and CINAHL databases. The following keywords were used: neoplasm, exercise and VO2max. Keywords were combined using the Boolean operators “AND” and “OR”.

### Study selection

The titles were initially read, and researchers later read the abstract and full documents. All duplicates and those that failed to meet the inclusion criteria were deleted.

The inclusion criteria were randomised control trial, control trial, human studies and language: English, Spanish, French and/or Portuguese.

All studies without oncology patients, TPE intervention and papers without changes in VO2max were excluded.

Two researchers with more than 10 years of experience in this area performed a literature search and study selection.

### Study of variables

The structural characteristics of the selected articles were studied, along with the other principal variables.

The articles’ structural characteristics can be found in Table 1. Tumour type, gender and age of participants were all studied. Types of training are also shown, as well as some important factors in prescribing a TPE programme: exercise type, session time, duration and supervision.

**Table 1:** PEDro scale

	*Type of study*	*Randomisation*	*Concealed allocation*	*Groups similar baseline*	*Blinding of subjects*	*Blinding of therapist*	*Blinding of assessors*	*85% n*	*Intention to treat*	*Between-group*	*A point measure*	*Score PEDro*
*Alizadeh *[39]	RCT	X	X	X	X	X	X	X	X	X	X	**10**
*Bhatia *[31]	RCT	X	-	-	X	-	-	X	X	X	X	**6**
*Casla *[27]	RCT	X	X	-	X	-	-	X	X	X	X	**7**
*Courneya *[37]	RCT	X	-	X	-	-	-	X	X	X	X	**6**
*de Paulo *[20]	RCT	X	X	-	-	-	-	-	X	X	X	**5**
*Dieli-Conwright *[28]	RCT	X	X	X	-	X	-	-	X	X	X	**7**
*Dieli-Conwright *[26]	RCT	X	X	X	X	X	-	-	X	X	X	**8**
*Duggan *[30]	RCT	X	X	X	-	-	-	X	X	X	X	**7**
*Edvardsen *[35]	RCT	X	X	X	X	-	-	X	X	X	X	**8**
*Grabenbauer *[36]	CT	X	-	-	-	-	-	-	X	X	X	**4**
*Grote *[9]	CT	X	-	-	-	-	-	-	X	X	X	**4**
*Hannam Arem *[29]	RCT	X	X	X	X	X	-	-	X	X	X	**8**
*Hvid *[12]	CT	-	-	X	-	-	-	X	X	X	X	**6**
*Lahart *[40]	RCT	X	X	X	X	X	-	-	X	X	X	**8**
*Lee *[18]	RCT (pilot)	X	X	X	X	X	-	-	X	X	X	**8**
*Neill *[10]	RCT	X	X	X	X	X	X	X	X	X	X	**10**
*May *[32]	RCT	X	X	-	X	-	-	X	X	X	X	**7**
*Mcneil *[22]	RCT	X	X	X	X	X	-	-	X	X	X	**8**
*Mehnert *[21]	RCT	X	X	X	X	X	X	X	X	X	X	**10**
*Quist *[33]	RCT	X	-	-	-	-	-	-	X	X	X	**4**
*Scott *[34]	RCT	X	X	X	X	X	X	X	X	X	X	**10**
*Adams *[13]	RCT	X	X	-	X	-	X	X	X	X	X	**7**
*Tosti *[19]	CT	X	-	-	X	-	-	-	X	X	X	**5**
*Uth *[14]	RCT	X	X	X	X	-	X	X	X	X	X	**9**
*Wall *[15]	RCT	X	-	-	-	-	-	X	X	X	X	**5**
*Yee *[41]	RCT (pilot)	X	X	X	X	X	-	-	X	X	X	**8**
*Zhijun *[42]	RCT	X	-	X	-	-	-	X	X	X	X	**6**

Abreviattions : *RCT* Randomiced control trial, *CT* Control trial

The principal variable is subjects’ VO2max. The changes after intervention with a TPE programme in oncology patients were highlighted.

PEDro scale was used to assess study methodology.

This scale consists of 10 points (selection criteria; randomisation of selection: hidden allocation; initial comparability between groups; all blinded subjects; all blinded therapists; all blinded evaluators; adequacy of follow-up; conduct of the analysis with treatment intent; comparison of results between groups; the existence of point and variability measures) that may be dichotomous Yes and No response, granted based on compliance or non-compliance with the requirements of the particular point.

Those studies with scores over or similar to 6 points were considered high quality, and those studies with scores below 6 points were considered low quality.

### Statistical analysis

To determine the overall OR of the selected studies, the Meta-DiSc software was used. Cochran’s Q test was used to evaluate statistical heterogeneity and the forest plot was developed with the same software. The heterogeneity of the studies was then calculated. To calculate heterogeneity, the I2 statistic was calculated. The following scale was used to stratify the different heterogeneity values [11]: large heterogeneity I2 > 50%; moderate heterogeneity I225–50%; low heterogeneity: I2 < 25%; and null heterogeneity I2 = 0. The level of significance was *p* ≤ 0.05.

## Results

After searching the literature in principal databases using the keywords, a total of 239 documents were obtained. Fifteen studies were excluded due to duplication, and the 224 remaining documents were subject to inclusion criteria. After applying inclusion criteria and reading titles and abstracts, 208 documents were later eliminated.

**Fig. 1:**
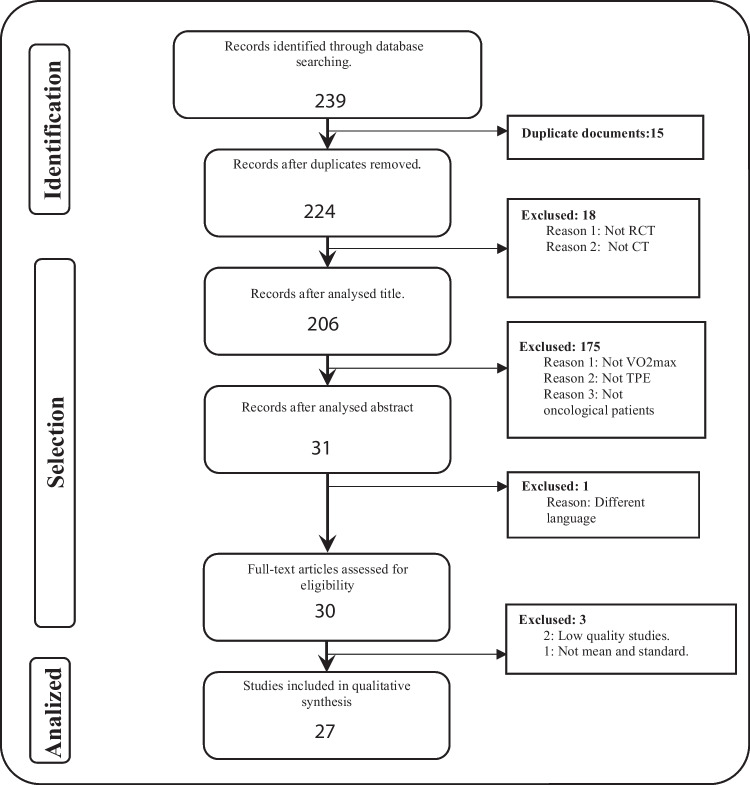
Flowchart of search results and filtering of the documents selected

Finally, those documents with a different language to the inclusion criteria were excluded. Papers with low internal validity were also excluded. The present review analysed a total of 28 articles. For more details of the search results, see Fig. 1.

The articles’ structural characteristics are shown in Tables 2, 3 and 4. These tables show the fundamental factors to prescribe a TPE programme. A total of 2558 patients aged between 18 and 80 were studied in this review. All documents contemplated primary tumour and/or secondary tumour. The most repeated type of training was combined training. The frequency ranged between 2 and 7 days per week. Session time ranged from 30 to 90 min. Accumulated exercise time of the week oscillated between 70 and 220 min per week. The programme’s duration ranged between 25 days and 48 weeks. Twenty-two articles of the total 28 selected offered a TPE programme supervised by a health professional.

**Table 2:** Structural characteristics of the selected articles. “Cancer men”

	TYPE OF STUDY	TYPE OF CANCER	PATIENTS	AGE	TYPE TRAINING	TIME/WEEK	DURATION OF SESSION FREQUENCY	DURATION OF THE PROGRAM	SUPERVISED EXERCISE TIME
HVID [12]	RCT	Prostate	21	67.8 (6.4)	Aerobic training	105 min. /week	35 min 3 d/week	12 weeks	12 weeks
ADAMS [13]	RCT	Testicular	63	18–80	High intensity interval training	105/week	3 d/week 35 min	12 weeks	12 weeks
UTH [14]	RCT	Prostate cancer	57	43–74	Football	180/week	3 d/week 60 min	12 weeks	12 weeks
WALL [15]	RCT	Prostate cancer	265	69.1	Combined training	270 min /week	2 d/week 60 min	24 weeks	24 weeks

Abbreviations: *RCT* randomised controlled trial

**Table 3:** Structural characteristics of the selected articles. “Cancer women”

	TYPE OF STUDY	TYPE OF CANCER	PATIENTS	AGE	TYPE TRAINING	TIME/WEEK	DURATION OF SESSION FREQUENCY	DURATION OF THE PROGRAM	SUPERVISED EXERCISE TIME
**ALIZADEH **[39]	RCT	Breast cancer	456	48.42 (7.54) Mean (SD)	Combined training	114 min/week	3 d/week 38 min	12 weeks	12 weeks
**CASLA **[27]	RCT	Breast cancer	235	18–70	Combined training	-	2 d/week	12 weeks	12 weeks
**DE PAULO **[20]	RCT	Breast cancer	313	63.6 (7.2) Mean (SD)	Combined training	210–270 min /week	3 d/week 70–90 min	36 weeks	36 weeks
**DIELI-CONWRIGHT **[28]	RCT	Breast cancer	100	53.5 (10.4) Mean (SD)	Combined training	-	3 d/week	16 weeks	16 weeks
**DIELI-CONWRIGHT **[26]	RCT	Breast cancer	418	-	Combined training	150–240 min/week	50–80 min 3 d/week	16 weeks 12 weeks tracking	16 weeks
**DUGGAN **[30]	RCT	Breast cancer	173	50–75	Aerobic training	225 min/week	45 min 5 d/week	48 weeks	
**HANNAM A**rem [29]	RCT	Breast cancer	121	Postmenopausal women	Combined training		2 d/week	48 weeks	48 weeks
**LAHART **[40]	RCT	Breast cancer	150	18–72	Aerobic training	90–210 min/week	3–7 d/week 30 min	24 weeks	12 weeks
**LEE **[18]	RCT (pilot)	Breast cancer	58	46.9 (9.8) Mean (SD)	High intensity interval training		3 d/week 30 min	8 weeks	8 weeks
**MCNEIL **[22]	RCT	Breast cancer	109	18	Aerobic training		150–300 min/week	24 weeks	
**MEHNERT **[21]	RCT	Breast cancer	156	18–54	Combined training		2 d/week	10 weeks	
**SCOTT **[34]	RCT	Metastatic breast cancer	65	21–80	Aerobic training		3 d/week	12 weeks	12weeks
**TOSTI **[19]	CT	Breast cancer	X	25–75	Resistance training				
**YEE **[41]	RCT (pilot)	Metastatic breast cancer	18	›18	Combined training	80–110 min/week	2 d/week 40–55 min	8 weeks	8 weeks
**ZHIJUN **[42]	RCT	Breast cancer	70	43.1 (5.4)	Aerobic training		50 min	16 weeks	16 weeks

Abbreviation: *RCT* randomised controlled trial, *CT* control trial

**Table 4:** Structural characteristics of the selected articles. “Cancer men and women”

	TYPE OF STUDY	TYPE OF CANCER	PATIENTS	AGE	TYPE TRAINING	TIME/WEEK	DURATION OF SESSION FREQUENCY	DURATION OF THE PROGRAM	SUPERVISED EXERCISE TIME
BHATIA [31]	RCT	Lung	151	64	High intensity interval training	90 min/week	30 min 3 d/week	Mean 25 days	25 days
COURNEYA [37]	RCT	Colon	211	65	Aerobic training	x	x	3 years	48 days
EDVARDSEN [35]	RCT	Lung	106	64.6	Combined training	240 min/week	60 min 3 d/week	20 weeks	20 weeks
GRABENBAUER [36]	CT	Breast cancer Colorectal cancer Endometrial cancer Brain tumour Lymphoma	52	›18	X	90–180 min/week	30–60 min 3 d/week	12 months	12 weeks
GROTE [9]	CT	Cancer survivors	76	X	Combined training	180 min/week	60 min 3 d/week	26 weeks	
NEILL [10]	RCT	Esophagogastric cancer	45	67.19	Combined training	210 min/week	35 min 6 d/weeks	12 weeks	10 weeks
MAY [32]	RCT	Cancer survivors	176	48.8	Combined training	260 min/week	120 min 2 d/week	12 weeks	12 weeks
QUIST [33]	RCT	Breast cancer Gynecological cancer Others	70	18–65	Combined training	270 min/week 120 min/week	3 d/week 90 min 4 d/week (low intensity) 30 min	6 weeks	

Abbreviations: *RCT* randomised controlled trial, *CT* control trial

**Fig. 2:**
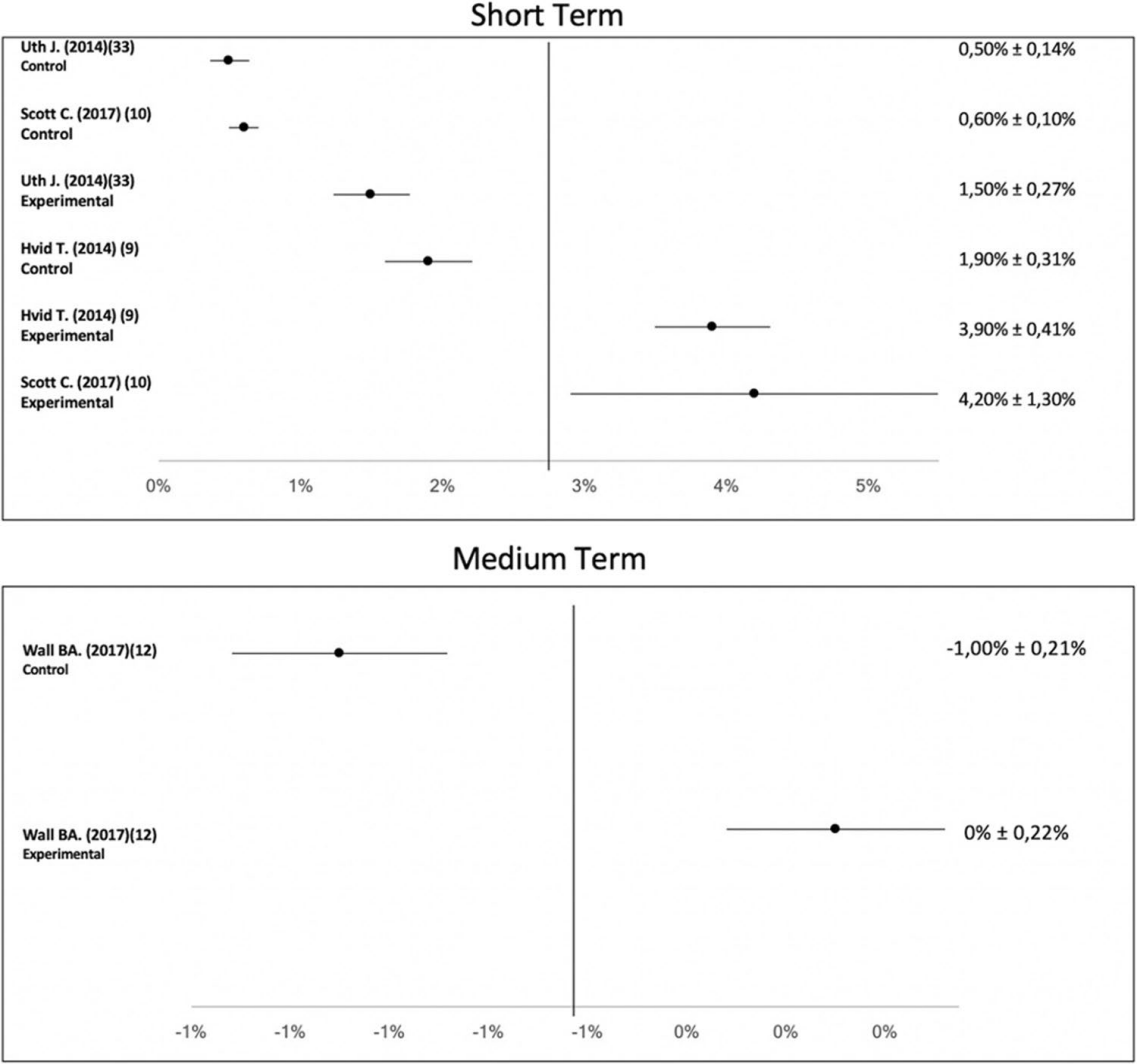
Forest plot of the effect on VO2max in male cancer patients in the short and medium term

**Fig. 3:**
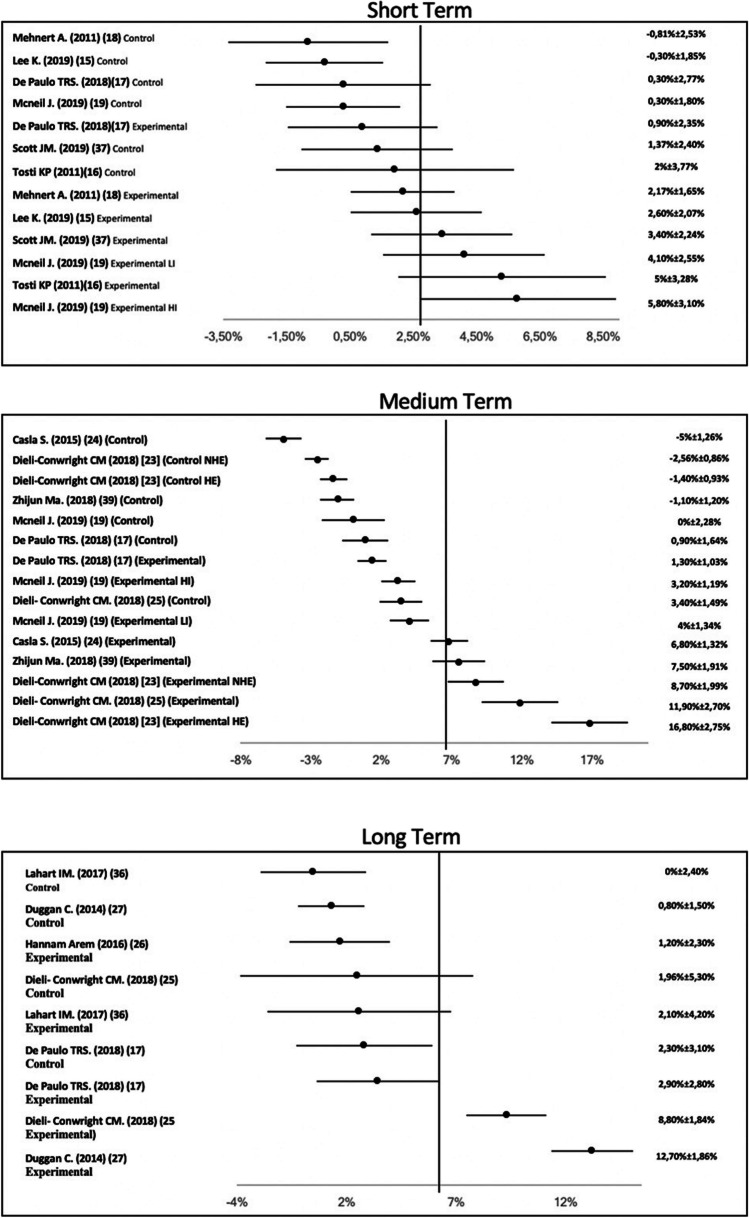
Forest plot of the effect on VO2max in female cancer patients in the short, medium, and long term

The results of the meta-analysis and the forest plot are shown in Figs. 2 and 3. The heterogeneity of the effect sizes of the studies selected for the meta-analysis observed values of Q = 35.47 and df = 14. Furthermore, the results observed in the other heterogeneity test presented a moderate value (I2 = 33%). Specifically, Fig. 2 shows the values obtained in the analysis carried out on men’s cancer, both in the short and medium term. None of the included studies analysed the long-term effect of the intervention. The improvement ranges between 1.5 and 4.2% in male-associated cancer.

On the other hand, Fig. 3 shows the short-, medium- and long-term results of the effect of the intervention on female cancers. The articles selected have used different measurement instruments, but the most repeated were the Bruce Protocol and Treadmill Walking Test. Vo2max in female-associated cancer was measured at baseline, pre- and post-intervention. Vo2max has a range between − 0.3 and 53% in the experimental group.

**Fig. 4:**
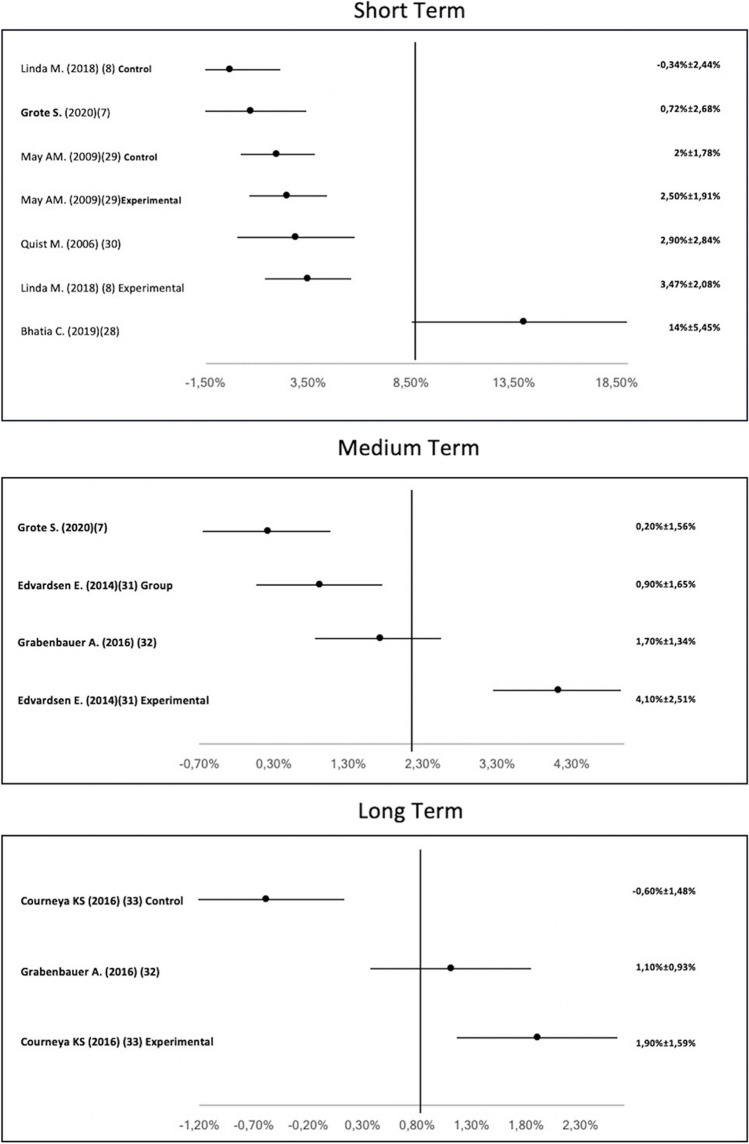
Forest plot of the effect on VO2max in female-male cancer patients in the short, medium, and long term

Figure 4 shows Vo2max with the control group mean and case group mean. This variable is shown pre- and post-intervention. The cardiopulmonary exercise test is the most repeated measurement instrument amongst studies. Vo2max in men’s and women’s cancer was measured in baseline, short term, medium term and long term. The improvement ranges between − 0.72 and 14%.

## Discussion

The main aim of this review was to analyse if VO2max in cancer patients increases after completing a TPE programme. The VO2max of the 2558 patients studied presents some variations by gender, intervention and moment of measurement. For male-associated cancer, the increased VO2max ranged between 1.5 and 4.2% after the intervention. With regard to diverse types of tumours related to women, the variable VO2max was measured in the short, medium and long term. The increase in VO2max in female-associated cancer was between 0.3 and 53% in the short term. In the medium term, it was between 0.9 and 16.8%, and in the long term between 2.9 and 12.7%. Types of cancer affecting both genders presented an oscillation between 2 and 3.47% in the short term, 0.5 and 4.1% in the medium term and 1.1 and 1.9% in the long term after intervention with the TPE programme.

### Vo2max “men and cancer”

Following analysis of four studies of male-associated tumours, three studies [12, 13, 14] show an increase between 1.5 and 4.2% in VO2max consumption in the short term after intervention. In the medium term, the VO2max results for prostate cancer survivors did not show an increase in the experimental group after intervention [15]. In the short term, the optimal frequency to generate an increase in VO2max in men with prostate and testicular cancer is 3 days a week [12, 13, 14], with a duration per session equal to or longer than 35 min and with a minimum duration of the intervention protocol of 12 weeks and, preferably, supervised [12, 13, 14]. Although, analysing the data well, it is also observed that the weekly intervention time should not be less than 105 min, there is a lack of studies that analyse how the intervention time should be distributed. In men with type II diabetes [16] and healthy men [17], participants’ VO2max increases with a frequency of 3 days per week. This increase in healthy men is 4.8% [17], which is better than the increase for oncology patients. Similarly to healthy men [17], high-intensity interval training (HIIT), supervised 3 days per week, achieves a higher increase in VO2max in men with prostate and testicular cancer [13]. This increase is 4.2% [13]. In the short term, all studies analysed [12, 13, 14] present the same frequency (3 days per week), and these studies have a supervised intervention. In the non-oncology population, frequency and supervision of intervention are similar to the studies analysed [16, 17]. In addition, it is important to remark a minimum of training time per week. As to intensity of intervention, the authors believe it should be moderate–high intensity [13, 17]. With high-intensity training, the authors obtain better results both in oncology patients [13] and in healthy subjects [17]. There are discrepancies as to the type of training, as well as the duration of the TPE programme. Future studies will be necessary to identify the most suitable type of training, as well as the most suitable duration of intervention to obtain a significant increase in male-associated VO2max. In the medium term, VO2max is measured by only one analysed study [15], and this measurement does not vary after the intervention. This intervention presents a frequency of 2 days per week, is medium–low intensity and is supervised [15]. The frequency and intensity of this intervention are lower than other interventions studied [12, 13, 14, 16, 17]. These factors may be the cause of the variable (VO2max) not showing an improvement.

### Vo2max “women and cancer”

The results offered by 15 documents which analyse the change in VO2max in tumours associated with women are very disparate in the three measurement times (short term, medium term and long term). In the short term, the oscillation range of VO2max is between − 0.3 [18] and 53% [19]. Analysed studies have used combined training [20, 21], HIIT [18, 22] and strength training [19]. The best improvement is obtained with strength training (55%) [19]. An intervention with HIIT [18] generates a deterioration in the experimental group, but this deterioration is less than for the control group. Despite these negative results [18], the deterioration suffered by the control group is higher. These results reaffirm that doing TPE is a better option than not doing it in order to maintain cardiorespiratory function. Session time along with programme duration of this intervention [18] is lower than those proposed by other authors [19, 20, 21, 22]. These factors may be the cause of deteriorating Vo2max in participants [18]. The TPE programme that generates a clinically relevant improvement in VO2max in female cancer sufferers is the 24-week programme [22]. The 24-week programme also generates a significant improvement in VO2max in women with fibromyalgia after intervention (17%) [23]. The analysed studies offer a frequency between 2 and 3 days per week [18, 20, 21]. The external literature offers the same frequency in postmenopausal women [6], women with fibromyalgia [23], obese women [24] and postmenopausal women [25]. There is controversy about some important factors in planning TPE programmes (intensity and type training) in women with cancer. This controversy is due to the lack of information regarding intensity, as intensity of intervention is offered by only two [18, 22] to five studies analysed. The controversy is also due to the type of training, as the authors use four different types in their interventions. Future studies will be necessary to identify the type of training, as well as the intensity of training suitable for tumours associated with women.

In the medium term, all interventions show improvements in the VO2max of their participants, oscillating between 0.9 [20] and 16.8% [26]. It would seem that, in the medium term, the type of training that generates major benefits in VO2max is combined training (between 0.9 and 16.8%). This type of training is used by five to seven studies analysed [20, 26, 27, 28, 29]. There is a slight controversy as to the supervision of TPE programme, as the intervention proposed by Mcneil [22] is not supervised, and this intervention shows an increase between 3.2 and 4% in the VO2max of participants. These results are lower than those observed in supervised interventions, which show improvements that oscillate between 0.9 [20] and 16.8% [28]. Furthermore, the improvement in participants in the programme proposed by Mcneil [22] shows a deterioration in the medium term, which may be due to the non-supervision of the intervention, as the rest of the interventions are supervised and their improvement in VO2max is maintained over time [20]. As is the case with female cancer sufferers [25], the supervised intervention for postmenopausal women shows an increase of 6.4% in the VO2max of participants. In obese breast cancer survivors [24], the improvement is more discreet after the supervised intervention, and corporal mass index could be an influential factor in the improvement of VO2max. The frequency of the intervention in postmenopausal [25] women and obese breast cancer survivors [24] is 2–3 days per week. This frequency is proposed by the majority of authors to increase VO2max in cancer sufferers [20, 26, 27, 28, 29]. It appears that frequency and supervision are two influential factors in planning a TPE programme in women with cancer in order to achieve an improvement in their VO2max. In 2019, The American Cancer Society [7] updated a review with more than 13,000 cancer patients, including breast cancer sufferers. This review shows that more than 75% of programmes are supervised by a health professional [7].

In the long term, only 4 of the 15 studies analysed offer measurements [20, 27, 28, 30]. VO2max shows an improvement between 2.9 and 12.7% after intervention with TPE. The type of training most repeated by the authors is combined training [20] [27] [28], although the major increase in VO2max (12.7%) is obtained with aerobic training [30]. In a similar population, the intervention with postmenopausal women [6] is also with aerobic training, which could be the most suitable to increase VO2max in women in the long term. The disparity in the four studies analysed [20, 27, 28, 30] may be due to two factors: time of session and duration of the programme. The authors propose a time of session between 38 [27] and 90 min [20]. The best increase in VO2max is obtained within 45 min of the session [30], and this increase generated an improvement of 12.7% in the vo2max of participants. With regard to the duration of the intervention, the authors propose a duration of between 12 [27] and 48 weeks [30]; a 48-week duration shows an increase of 12.7% [30], followed by an improvement of 8.8% for 16 weeks [28]. It would appear that a major increase in VO2max of the participants requires the intervention to last more than 12 weeks. It would be interesting for future studies to show all influential factors in a TPE programme (frequency, intensity, type of training, time of session and duration of intervention) to achieve a significant increase in VO2max in women in the long term.

### Vo2max “male and female cancer”

In the short term, five studies that analysed males and females with cancer present an increase in VO2max between − 0.75 [9] and 14% [31]. After a supervised HIIT in lung cancer patients, there is an improvement of 14% in VO2max [31]. However, the most repeated training is combined training, which shows an improvement of − 0.72% [9], 3.7% [10], 2.5% [32] and 2.9% [33]. Despite being the most repeated training, Grote [9] offers negative results after the intervention. Non-supervision could be the cause of a decrease in VO2max after the TAPE programme [9], as the other authors obtain improvement in VO2max after a supervised intervention [10, 31, 32]. In the short term, two influential factors in the increase in VO2max, both in the oncological population and in the healthy population, are supervision in the programme and the high intensity of the training. In cancer patients [24], active healthy population [17] and inactive healthy population [6], we can see that the supervised intervention brings better results in the increase in participants’ VO2max. A supervised intervention shows an increase of 0.1% in obese breast cancer survivors [34], an increase of 4.8% in healthy men [17] and an increase of 0.17% in postmenopausal women with low physical activity [6]. As with the oncological population [13], in the healthy population [17] [25], a major increase in VO2max is obtained with a high-intensity dose in training. With a high-intensity intervention, the authors obtain an increase of 6.4% in premenopausal women’s VO2max [25] and an increase of 4.8% [17] in healthy men. This factor (high intensity) is recommended to improve other adverse effects of cancer. “Exercise guideline for cancer survivors” [8] recommends high intensity in training to decrease anxiety, depression and lymphoedema. Training with high intensity also increases the quality of life and physical function of patients [8]*.* In the medium term, four studies take measurements after intervention, obtaining an improvement range in VO2max between − 0.2 [9] and 4.1% [35]. The most repeated training is combined training with a frequency of 3 days per week and 60 min per session [9, 10, 35]. All experimental group results are positive. In contrast, Grote [9] proposes an intervention with combined training, but this intervention produces a decrease of 0.2% in VO2max. As in the short term, this decrease in VO2max could be due to the non-supervised intervention, as TPE is most effective if it is supervised by a health professional [7]. In the medium term, the effectiveness of the supervision programme could also be observed in obese breast cancer survivors [24] and in low-level physical activity postmenopausal women [6]. In both interventions, VO2max is maintained between 0.1 and 17% [6, 24]. Another influential factor in the decreased VO2max of the experimental group in Grote’s intervention [9] is the duration of the intervention (26 weeks). This duration is infrequent, as it is 12 weeks or 12 months in both the oncological population [10, 36] and the non-oncological population [6, 24]. With regard to the most repeated frequency in the analysed studies, 3 days per week generates an increase in the VO2max of participants [9, 35, 36]. This frequency (3 days per week) is used in men with type II diabetes [16] and healthy subjects [6, 17, 25], and all these interventions show an increase in VO2max of their participants.

In the long term, only two studies analyse changes in VO2max [36, 37].Vo2max improves by 1.9% with a supervised intervention of 3 days per week [36], and with anaerobic training for 3 years, VO2max improves by 1.1% [37]. There is a disparity in influential factors in the TPE programme which have been proposed by the authors [36, 37]. However, the 12-month programme for cancer patients [36] is the same duration as the authors propose for low-level physical activity in postmenopausal women [6]. For this healthy population, the intervention consists of aerobic training, which is the same type of training as for colonic patients [37]. It would appear that aerobic training and 12 months of duration are two factors to produce an increase in VO2max in the long term [6, 36, 37]. It would be interesting for future studies to contemplate an evolution in the intervention in order to maintain a stimulus that generates changes in the patients.

## Clinical applicability

The studies with the best results show an increase in the participants’ VO2max of between 4.2 [13] and 53% [19]. All study populations present results in the short term [13, 19, 26, 30, 31], but only studies about women and cancer [19, 26, 30] show clinically relevant improvements in the medium and long term. These studies present some common influential factors in planning a TPE programme. The most repeated frequency is 3 days per week [26, 30, 31] and the mean duration of the session is 45 min [13, 26, 30, 31]. All authors propose vigorous intensity training [13, 19, 26, 30, 31]. With regard to the duration of the TPE programme, the most notable is 12 weeks [13, 26] and all authors supervise the intervention [38, 19, 26, 30, 31]. As for type of training, there is a major disparity due to diversity in the study population, but the most repeated type of training is HIIT [38, 31]. Of the studies with best results in VO2max, all participants accumulate a mean of 150 min of exercise per week [38, 19, 26, 30, 31]. Additionally, another variable that must be taken into account is the intensity of the exercise. In this sense, although few studies have made a direct comparison between the effect of the same intervention at different intensities, in the one that has done so, it has been observed how higher intensities achieve better results in increasing VO2max with respect to the lowest intensities [22].

It is important to highlight supervision of intervention to guarantee the safety and efficacy of the TPE programme, regardless of cancer type.

For male-associated cancer, the most suitable frequency will be 3 days per week with a minimum duration of 35 min. The intensity should be moderate-vigorous and the duration of the programme should be 12 weeks. As for type of training for men with testicular cancer, HIIT would be of particular interest, while for men with prostate cancer, the type would be aerobic training.

In female cancer types, the most repeated are breast cancer and metastatic breast cancer. In the short term, the most suitable type of training for breast cancer participants would be strength training. This shows improvements in VO2max in breast cancer participants. In the medium term, the type of training should be combined training (strength and aerobic training), 3 days per week, with moderate-vigorous intensity and a programme duration of 12 weeks. To maintain VO2max benefits in breast cancer sufferers in the long term, the training should be aerobic and the frequency 5 days per week with a programme duration of 48 weeks. In metastatic breast cancer women, the results of the studies offer an aerobic programme with medium–high intensity, 3 days per week and a programme duration of 12 weeks.

With regard to cancers associated with both genders, we note the third most frequent tumour in Spain in 2022, namely lung cancer. An HIIT would be interesting for this type of cancer, 3 days per week with a minimum duration of 30 min.

Other adverse effects can influence the improvement in VO2max in oncology patients. An exclusive TPE intervention might not resolve these effects. A multidisciplinary intervention with exercise, nutrition and psychology could contemplate all these adverse effects. For example, high corporal mass index, as well as emotional distress, can interfere with the improvement in VO2max in cancer patients who have been intervened with TPE. For head and neck cancer (HNC) patients with radiotherapy treatment [5], a nutritional and psychological education intervention shows numerous benefits. HNC patients improve their nutritional state, quality of life, weight, depression and the interruption periods of radiotherapy treatment [5].

The American Cancer Society [5] shows the recommendations for nutrition and physical activity in oncology patients. Experts recommend a combined intervention with both therapies to prevent a primary tumour for patients with oncology treatment and survivors [5]. A multidisciplinary intervention in oncology patients to achieve greater benefits would therefore be interesting.

## Strengths and weaknesses

Those studies published in five languages (English, Spanish, Italian, French and Portuguese) were selected, although there may be other papers which are not included in the present review. Despite the search having been carried out in four leading databases, there could be other studies in other databases which have not been included in the present review.

## Conclusions

TPE is an efficient intervention to improve VO2max in oncology patients. TPE intervention should be supervised by health professionals, regardless of gender and type of cancer. The results in this review show that a TPE intervention lasting 12 weeks, 3 days per week with a moderate-vigorous intensity, increases VO2max in oncology patients. However, future studies would be necessary to evaluate the type of training that is most adequate according to gender and type of cancer. Studies carrying out measurements in the medium and long term of VO2max in participants would also be necessary.

## Data Availability

Data are availability under request to the corresponding author.
